# Immunotherapy and the Sequence Relative to Survival Outcomes in SCLC: Analysis of the National Cancer Database

**DOI:** 10.3390/cancers18040567

**Published:** 2026-02-09

**Authors:** Dan Yao, Yinting Liu, Wenyao Yu, Sisi Zheng, Lujie Huang, Mengsi Cai, Yan Zhuang, Youwen He, Xiaoying Huang

**Affiliations:** 1 Division of Pulmonary Medicine, The First Affiliated Hospital, Wenzhou Medical University, Wenzhou Key Laboratory of Interdiscipline and Translational Medicine, Wenzhou Key Laboratory of Heart and Lung, Wenzhou 325000, China; yaodan@wzhospital.cn (D.Y.); zhengsisi@wmu.edu.cn (S.Z.);; 2 Division of Biostatistics, College of Public Health, University of Nebraska Medical Center, Omaha, NE 68198, USA; yiliu@unmc.edu; 3 Department of Mathematics, University of California, San Diego, CA 92093, USA; w6yu@ucsd.edu; 4 Division of Bioinformatics & Biostatistics, Duke University Medical Center, Durham, NC 27710, USA; yz933@duke.edu; 5 Department of Integrative Immunobiology, Duke University School of Medicine, Durham, NC 27710, USA

**Keywords:** small-cell lung cancer (SCLC), immunotherapy sequence, chemoradiotherapy (CRT), overall survival (OS)

## Abstract

Background: SCLC remains an aggressive malignancy with limited therapeutic progress over the past few decades. It remains unclear how the sequence of immunotherapy initiation influences overall survival (OS) in SCLC; we performed a population-based analysis using NCDB to evaluate its association with survival. Methods: SCLC patients in NCDB from 2016 to 2021 were identified to evaluate the impact of immunotherapy on OS. Among ES-SCLC patients, we conducted subsequent analyses to clarify the relationship between the sequence of immunotherapy initiation and OS in the context of chemotherapy and CRT. Results: Among 69,820 eligible patients, 9242 received CRT plus immunotherapy (CRT + IO), and 11,755 received chemotherapy plus immunotherapy (Chemo + IO). In the overall population, adding immunotherapy to chemotherapy or CRT was associated with modestly improved survival. In ES-SCLC, immunotherapy was associated with longer survival in both the Chemo and CRT cohort, while the addition of immunotherapy did not confer benefits in limited-stage SCLC (LS-SCLC). Within the ES-SCLC Chemo + IO cohort, altering the initiated immunotherapy interval (0–90 days) did not show any meaningful difference in survival. By contrast, in the CRT + IO cohort, survival showed benefit in a time-dependent pattern: patients who initiated immunotherapy within 4–7 days after CRT had a trend of survival, which was consistent with the proposed immune activation window. Conclusions: This real-world analysis suggests that immunotherapy was associated with longer survival in ES-SCLC, CRT + IO is associated with improved OS, with a more obvious survival benefit when immunotherapy is initiated within 4–7 days after CRT. These findings hint at the potential importance of immunotherapy initiation sequence and warrant further prospective validation.

## Simple Summary

Small-cell lung cancer (SCLC) is an aggressive malignancy, and long-term survival remains poor. Immunotherapy has recently been integrated into first-line treatment regimen alongside chemotherapy (Chemo) or chemoradiotherapy (CRT) for extensive-stage SCLC (ES-SCLC); however, the benefits of combined immunotherapy are derived from highly selective patient populations in controlled clinical trials, evidence from the real-world remains limited, and the effectiveness of the sequence of immunotherapy initiation across different treatments has not been clearly defined. Using data from the National Cancer Database (NCDB), our analysis showed that ES-SCLC patients who received immunotherapy in addition to standard therapy had improved survival. Among those treated with CRT, optimal outcomes were observed when immunotherapy was initiated within 4–7 days following CRT compared to earlier or delayed initiation. These findings suggest that the sequence of immunotherapy initiation administration may influence treatment efficacy in ES-SCLC. Prospective clinical studies are warranted to validate these observations and define the ideal sequence for immunotherapy initiation.

## 1. Introduction

SCLC is an aggressive neuroendocrine malignancy characterized by rapid proliferation, early systemic dissemination, and a high relapse rate. It accounts for approximately 13–15% of all lung cancer cases worldwide and up to 20% in China. Although SCLC initially demonstrates high sensitivity to platinum–etoposide-based chemotherapy, nearly all patients eventually experience disease relapse. Long-term survival remains poor, with a 5-year OS rate below 10% [1]. In contrast to the significant therapeutic advances achieved in non-small-cell lung cancer (NSCLC) in recent years, progress in the treatment of SCLC has remained limited [2,3,4,5].

Recently, phase III clinical trials—including RATIONALE-312 [6], ASTRUM-005 [7], IMpower133 [8], and CASPIAN [9]—have shown that the addition of programmed cell death protein 1 (PD-1) or programmed death-ligand 1 (PD-L1) inhibitors to chemotherapy confers a modest but statistically significant improvement in both OS and progression-free survival (PFS) compared with chemotherapy alone. As a result, the combination of immune checkpoint inhibitors (ICIs) with platinum-based chemotherapy has become a standard first-line treatment regimen for ES-SCLC [10]. However, the demonstrated benefits of combined immunotherapy are largely derived from highly selective patient populations in controlled clinical trials [11,12]. Evidence from the real-world applicability of these findings remains limited, and the effectiveness of the sequence of immunotherapy initiation across different treatments has not been clearly defined. Therefore, large-scale, population-based, real-world evidence is needed to evaluate the impact of these treatment strategies on survival outcomes in broader clinical practice.

Accumulating evidence suggests that the sequence of immunotherapy initiation relative to chemotherapy and radiotherapy may be a critical determinant of treatment efficacy [13,14]. Chemotherapy and radiotherapy can induce immunogenic cell death (ICD), leading to the release of tumor antigens and activation of innate immunity within days. This is followed by the clonal expansion of antigen-specific CD8^+^ T cells, during which immune checkpoint blockade may exert a maximal therapeutic effect by enhancing effector T-cell function and sustaining immune-mediated tumor control [15,16,17]. Preclinical models indicate that initiating ICIs within this defined “window of adaptive immune activation” results in enhanced tumor regression and prolonged survival. In contrast, administering immunotherapy either concurrently—before adaptive immune activation occurs—or after the window has closed may fail to optimally engage the immune response, resulting in diminished therapeutic efficacy [18].

Despite these mechanistic insights, the clinical implications of the sequence of immunotherapy initiation in SCLC remain poorly understood, and the association between the sequence of immunotherapy initiation and survival outcomes has not been well characterized in real-world populations. Therefore, we conducted a population-based analysis using NCDB to evaluate the relationship between the sequence of immunotherapy initiation and survival outcomes among patients with ES-SCLC receiving chemotherapy or CRT. The previous studies based on NCDB [19,20,21] in various malignant tumors provided objective information for the assessment of actual treatment effects and were helpful for conducting clinically significant comparisons among a large and diverse patient population. The aim of this study was to generate real-world evidence to inform the optimal treatment sequence of immunotherapy initiation and guide the design of future prospective clinical trials.

## 2. Materials and Methods

### 2.1. Data Source and Study Population

We conducted a retrospective cohort study using NCDB (NCDB Participant User File, 2022 release), a hospital-based registry that captures a majority of newly diagnosed cancers in the United States [22]. Patients with SCLC diagnosed between 2016 and 2021 were identified. Eligible patients were aged ≥18 years and received one of four first-course treatment strategies recorded in the NCDB. Records with missing age data were excluded from propensity-score modeling. The analysis was limited to four predefined treatment groups: chemotherapy alone (“Chemo only”), chemotherapy plus immunotherapy (“Chemo + IO”), concurrent chemoradiotherapy without immunotherapy (“CRT only”), and concurrent chemoradiotherapy with immunotherapy (“CRT + IO”). CRT was defined as chemotherapy and radiotherapy initiated on the same calendar day, consistent with the operational definition of concurrent treatment in the NCDB. OS was defined as the time from diagnosis to death or last follow-up, as captured in the NCDB follow-up fields. Limited-stage (LS) and extensive-stage (ES) disease were defined according to standard clinical criteria using NCDB staging elements; internal database field names are omitted to avoid ambiguity.

NCDB does not provide drug-level details regarding specific chemotherapy agents, dosing schedules, individual ICIs, or treatment-related adverse events, including immune-related toxicities, dose reductions, and treatment discontinuations. During the study period (2016–2021), first-line systemic therapy for SCLC in the United States predominantly consisted of platinum–etoposide-based chemotherapy, and immunotherapy primarily involved PD-1 or PD-L1 inhibitors approved for ES-SCLC. Accordingly, treatment groups cannot be defined by specific drugs or drug doses.

### 2.2. Variables and Baseline Covariates

A priori covariates included age (continuous), sex, race/ethnicity, insurance status, Charlson–Deyo comorbidity score, year of diagnosis (treated as a categorical factor), and treatment facility type. To minimize distortion from heavy missingness and extreme category imbalance, the TNM clinical stage group and M category and the NCDB “analytic stage group” were summarized descriptively but were not included in propensity-score models. Categorical variables followed NCDB coding, with “Unknown” retained as its own level.

### 2.3. Study Aims and Analytical Overview

Two complementary questions were addressed. First, we evaluated the incremental association of adding immunotherapy to standard backbones by comparing Chemo + IO vs. Chemo only and CRT + IO vs. CRT only. Each comparison was performed in the overall cohort and separately within LS and ES disease. Second, within ES disease, we examined whether survival varied by the interval between the start of immunotherapy and the immediately preceding modality. For patients treated with CRT + IO, we defined the interval as time from the start of chemotherapy and radiation start to immunotherapy start (CRT + IO). For patients treated with Chemo + IO, we defined the interval as time from chemotherapy start to immunotherapy start (Chemo + IO). Intervals were restricted to non-negative values within 0–90 days to represent clinically plausible sequencing. Time intervals were grouped using several clinically motivated schemes. Intervals within 28 days were prespecified a priori based on biological considerations related to chemotherapy- and radiotherapy-induced immune activation, whereas longer intervals were evaluated in sensitivity analyses to assess the robustness of observed associations. Specifically, we examined grouping schemes defined in days (d) as follows: (i) same-day initiation, 1–5 d, 6–13 d, 14–28 d, and 29–90 d; (ii) same-day initiation, 1–7 d, 8–14 d, 15–28 d, and 29–90 d; (iii) same-day initiation, 1–3 d, 4–7 d, 8–14 d, and 15–28 d; and (iv) same-day initiation, 1–3 d, 4–7 d, 8–28 d, and 29–56 d.

### 2.4. Propensity Scores (PSs), Weighting, and Diagnostics

**Figure 1:**
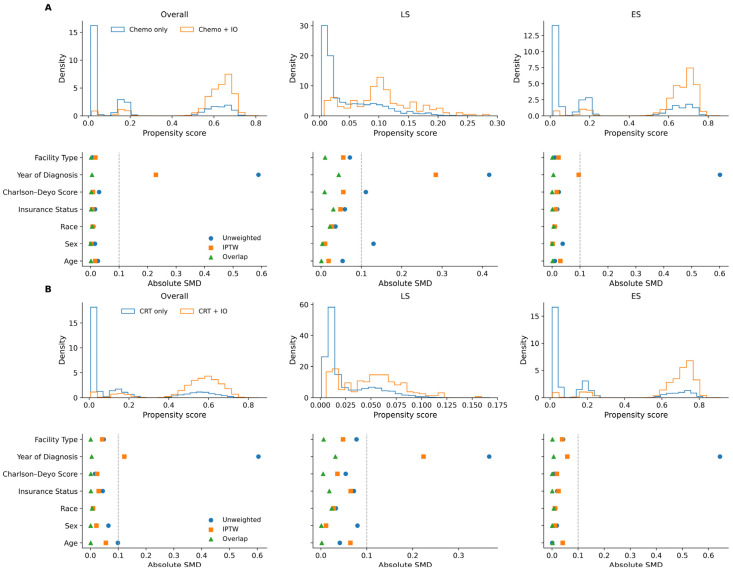
PS distributions and covariate balance for chemotherapy and chemoradiotherapy with or without immunotherapy under unweighted, IPTW, and OW. (**A**) PS diagnostics and covariate balance of Chemo vs. Chemo + IO in overall, LS-SCLC and ES-SCLC cohort. (**B**) PS diagnostics and covariate balance of CRT vs. CRT + IO in overall, LS-SCLC and ES-SCLC cohort.

For each pairwise comparison, we estimated PSs using logistic regression with one-hot encoding for categorical covariates (age, sex, race/ethnicity, insurance, Charlson–Deyo, year of diagnosis, and facility type). Two weighting strategies were derived from the fitted PS. Stabilized inverse probability of treatment weights (IPTW) were applied to balance measured baseline covariates between treatment groups, creating a weighted pseudo-population in which covariate distributions were comparable across treatment groups; extreme IPTW values were truncated at the 99th percentile. Overlap weights (OW) were additionally calculated to emphasize the region of common support [23]. For the ES sequence of immunotherapy initiation analyses, where treatment has more than two levels (the interval groups), PSs were obtained from a multinomial logistic regression using the same covariates. From these, we generated stabilized IPTW for the observed sequence of immunotherapy initiation group with truncation at the 99.5th percentile and OW tailored to the multigroup setting. Covariate balance was assessed using absolute standardized mean differences (ASMDs). For continuous variables, we used the difference in (weighted) means relative to the pooled (weighted) standard deviation (SD); for categorical variables, we averaged the ASMD across indicator levels. An ASMD below 0.10 was considered acceptable. We present diagnostic panels showing propensity-score distributions and ASMDs before and after weighting (Figure 1).

### 2.5. Survival Analyses

Unadjusted survival was described using Kaplan–Meier (KM) curves with log-rank tests. Weighted KM curves were then produced under IPTW and OW. For pairwise contrasts, we fit Cox proportional hazard (PH) models with a binary treatment indicator and report hazard ratios (HRs) with 95% confidence intervals (CIs). For multigroup sequence of immunotherapy initiation analyses, we fit Cox models with the sequence of immunotherapy initiation group as a categorical predictor (reference = same day, unless otherwise stated) and report HRs and 95% CIs for all comparisons, alongside global *p* values from a weighted log-rank or weighted Cox likelihood ratio test, as appropriate. Numbers at risk were displayed at 0, 12, 24, 36, 48, 60, and 72 months; for weighted panels, these counts represent sums of weights.

To quantify absolute survival benefit between sequence of immunotherapy initiation groups, we additionally summarized group differences using restricted mean survival time (RMST). RMST was estimated from the corresponding KM. For each sequence scheme, we calculated RMST for every sequence category and reported RMST differences relative to the reference category (same day). RMST analyses were conducted under unweighted, IPTW, and OW settings, consistent with the KM and Cox modeling framework above, and were visualized as RMST difference curves across τ to facilitate comparison of absolute gains or losses over clinically relevant follow-up horizons.

### 2.6. Descriptive Tables and Statistical Tests

Baseline characteristics for the four treatment arms were summarized overall (and, when indicated, within LS and ES). Continuous variables are shown as mean (SD) and compared using the Kruskal–Wallis test; categorical variables are shown as *n* (percent) and compared using the chi-square test. In the pairwise “Table 1” and “Table 2” outputs, we report unweighted and weighted cell counts/percentages (the denominator for weighted percentages is the sum of weights) together with ASMDs to document balance.

### 2.7. Data Handling and Missingness

Age was coerced to numeric; records with missing age were excluded from propensity-score modeling. For survival analyses, times were required to be non-negative, and events were constrained to binary values. For weighted analyses, observations with missing, infinite, or non-positive weights after PS estimation were excluded. Categorical maps were applied to produce human-readable levels; “Unknown” was retained.

### 2.8. Software

Analyses were performed in Python (versions 3.10.4) using pandas (v2.3.3) for data processing, scikit-learn (v1.2.2) for propensity-score estimation, and lifelines (v0.30.0) for Kaplan–Meier and Cox modeling. Two-sided *p* values <0.05 were considered statistically significant.

### 2.9. Reporting Conventions

Balance was judged by ASMD (<0.10 acceptable). We report HRs with 95% CIs and *p* values from standard log-rank tests (unweighted), from multigroup weighted log-rank tests (sequence panels), or from likelihood ratio tests in weighted Cox models when appropriate. Weighted numbers at risk and percentages reflect sums of weights rather than raw counts [24].

## 3. Results

### 3.1. Study Population

**Figure 2:**
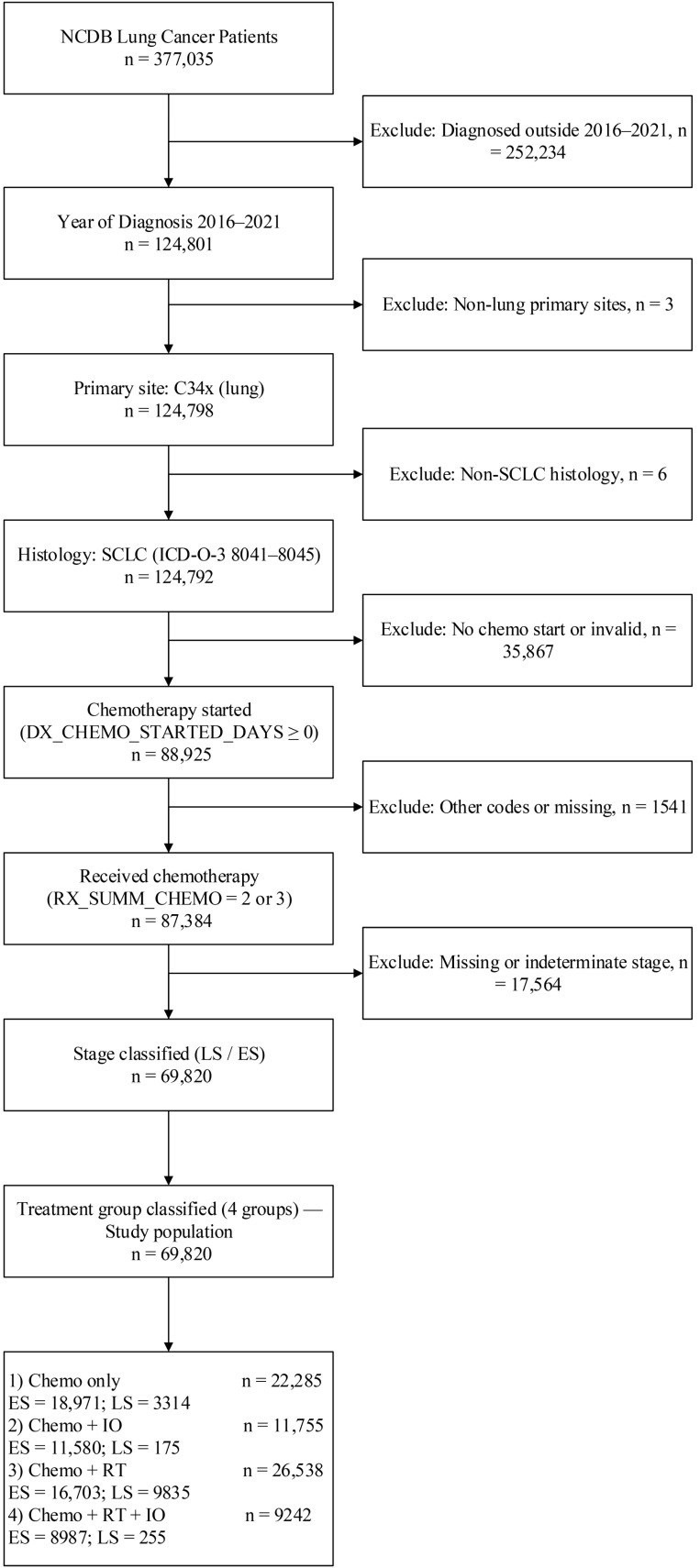
This diagram illustrates the study population selection and exclusion criteria.

The study cohort and treatment groups are summarized in Figure 2, which outlines the inclusion and exclusion process and final group allocation. From a total of 377,035 lung cancer records in NCDB, we restricted the analysis to cases diagnosed between 2016 and 2021, with primary lung tumors, confirmed small-cell histology, and a valid chemotherapy initiation date. After excluding NSCLC, cases with missing or invalid treatment dates, and those without a defined stage category, 69,820 patients were included in the final analysis. Patients were classified into four mutually exclusive treatment groups: chemotherapy alone (*n* = 22,285), chemotherapy plus immunotherapy (Chemo + IO; *n* = 11,755), chemoradiotherapy alone (CRT only; *n* = 26,538), and chemoradiotherapy plus immunotherapy (CRT + IO; *n* = 9242). The distribution of LS and ES disease within each group was as follows: chemo only (ES = 18,971; LS = 3314), Chemo + IO (ES = 11,580; LS = 175), CRT only (ES = 16,703; LS = 9835), and CRT + IO (ES = 8987; LS = 255).

### 3.2. Survival Effects of Adding Immunotherapy

**Figure 3:**
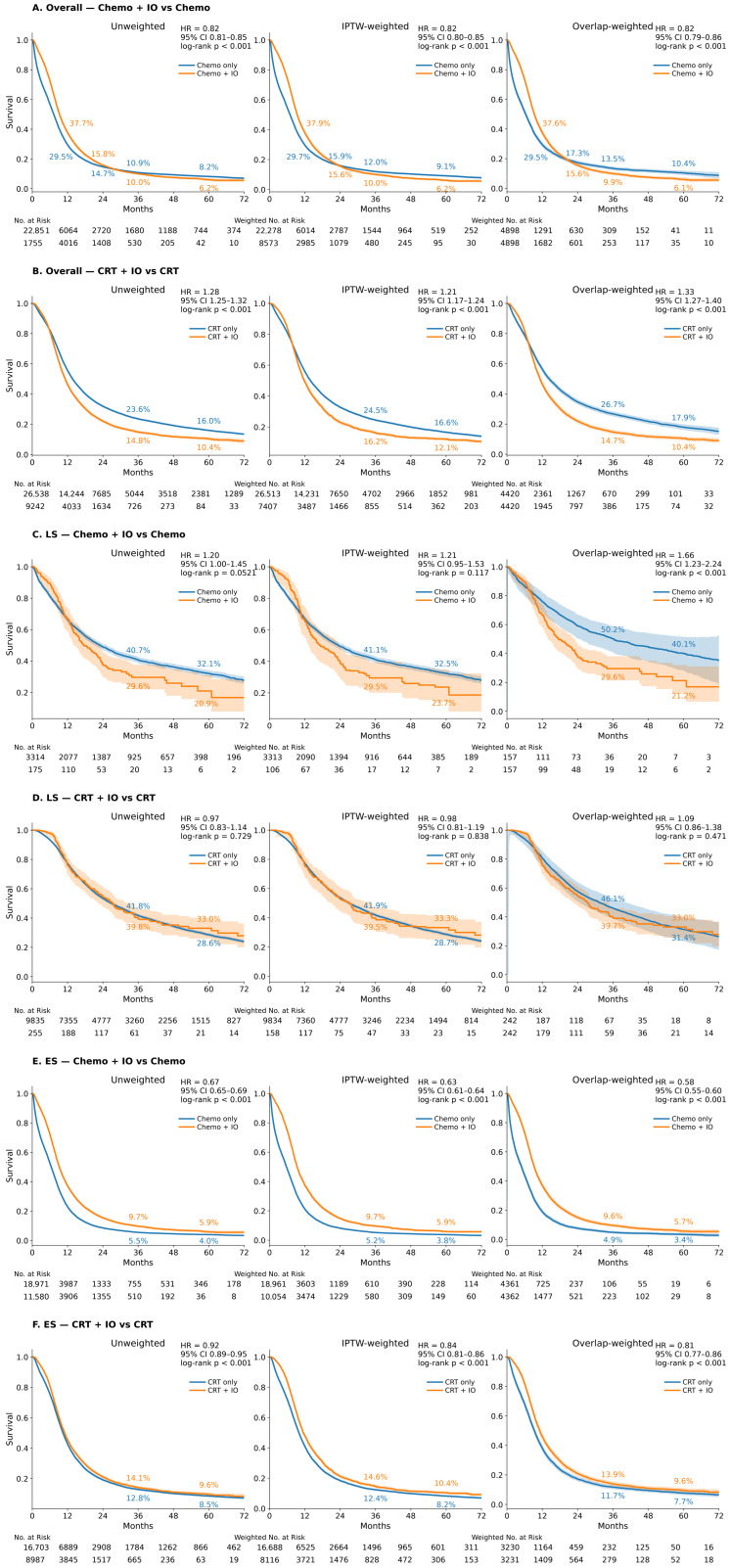
Survival analysis of CRT and Chemo, with or without immunotherapy in overall, LS-SCLC and ES-SCLC cohort: unweighted, IPTW, and OW Kaplan–Meier curves. (**A**). Survival analysis of chemotherapy vs. Chemo + IO in the overall cohort. (**B**). Survival analysis of CRT vs. CRT + IO in the overall cohort. (**C**). Survival analysis of chemotherapy vs. Chemo + IO in LS-SCLC cohort. (**D**). Survival analysis of CRT vs. CRT + IO in LS-SCLC cohort. (**E**). Survival analysis of chemotherapy vs. Chemo + IO in ES-SCLC cohort. (**F**). Survival analysis of CRT vs. CRT + IO in ES-SCLC cohort.

In the overall population, the addition of immunotherapy to either chemotherapy or CRT did not show a clinically meaningful survival advantage, with survival curves showing minimal divergence under both IPTW and overlap weighting (Figure 3A,B). In patients with LS-SCLC, immunotherapy added to chemotherapy was associated with inferior survival, whereas no meaningful difference was observed when immunotherapy was combined with CRT (Figure 3C,D). Among patients with ES-SCLC, the Chemo + IO group demonstrated improved Kaplan–Meier survival compared to chemotherapy alone in both unweighted analyses and after IPTW and OW (Figure 3E). The magnitude of effect was consistent across analytical methods (hazard ratios from Cox models ranging from approximately 0.58 to 0.67 across unweighted, IPTW, and OW analyses, with concordant log-rank test *p* values), and absolute survival advantages were maintained at 36 and 60 months. A similar trend was observed when immunotherapy was added to CRT: CRT + IO showed superior outcomes relative to CRT alone, with consistent hazard attenuation across weighting approaches (HRs ~0.80–0.83; Figure 3F).

In LS-SCLC, no significant survival benefit associated with immunotherapy was observed. The relatively small number of patients receiving immunotherapy in this subgroup resulted in unstable estimates and wide CIs. Furthermore, clinical evidence supporting the efficacy of immunotherapy in this subgroup remains insufficient, as phase III trials establishing immunotherapy benefits were primarily conducted in patients with ES disease. Therefore, subsequent analyses were restricted to the ES-SCLC cohort to assess whether the sequence of immunotherapy initiation relative to chemotherapy or CRT was associated with survival outcomes.

### 3.3. Baseline Covariate Balance in ES-SCLC

Among patients with ES-SCLC, the four treatment groups were: chemo only (*n* = 18,971), Chemo + IO (*n* = 11,580), CRT alone (*n* = 16,703), and CRT + IO (*n* = 8987). Mean age was comparable between the chemo-only and Chemo + IO groups (67.63 vs. 67.54 years; ASMD 0.026; IPTW ASMD 0.018; overlap weighting ASMD < 0.001) and similarly balanced between CRT + IO and CRT alone (64.91 vs. 64.91 years; ASMD 0.010; IPTW ASMD 0.028; and overlap weighting ASMD < 0.001). Sex distribution was nearly balanced, with minimal differences between groups (chemotherapy pair ASMD 0.015; IPTW ASMD 0.002; overlap weighting ASMD < 0.001; CRT pair ASMD 0.038; IPTW ASMD 0.002; and overlap weighting ASMD < 0.001). Race/ethnicity was predominantly White (~89% across all arms), with smaller proportions of Black and other racial/ethnic groups; balance was excellent (all ASMDs ≤ 0.01). Insurance type showed modest imbalances in the unweighted data, with Medicare coverage more prevalent in chemotherapy groups and private insurance more common in CRT groups, although these differences were attenuated after weighting. Charlson–Deyo comorbidity scores were well balanced across arms (ASMDs ≤ 0.050). The year of diagnosis exhibited the greatest initial imbalance due to increasing immunotherapy utilization after 2019 (unweighted ASMD: chemotherapy pair 0.589, CRT pair 0.601), which was substantially reduced after weighting, particularly using OW (chemotherapy pair ASMD 0.005, CRT pair ASMD 0.005). Facility type distributions were similar across groups, with minor imbalances (most ASMDs were ≤ 0.01, except for slight deviations under IPTW). Overall, covariate balance after weighting was acceptable, with all overlap-weighted ASMDs and most IPTW-weighted ASMDs below 0.10 (Table 1: Chemo only vs. Chemo + IO; and Table 2: CRT only vs. CRT + IO). The only notable exception was the year of diagnosis under IPTW, where moderate imbalance persisted (ASMD 0.221 for chemotherapy and 0.095 for CRT), reflecting the rapid uptake of immunotherapy in more recent years.

### 3.4. Survival in Different Sequence of Chemotherapy Plus Immunotherapy

Given the consistent survival advantage associated with immunotherapy, we further investigated whether the sequence of immunotherapy initiation relative to the preceding treatment modality influenced outcomes in patients with ES-SCLC. Two clinically distinct treatment sequences of immunotherapy initiation were analyzed using non-negative intervals restricted to 0–90 days: Chemo + IO and CRT + IO.

**Figure 4:**
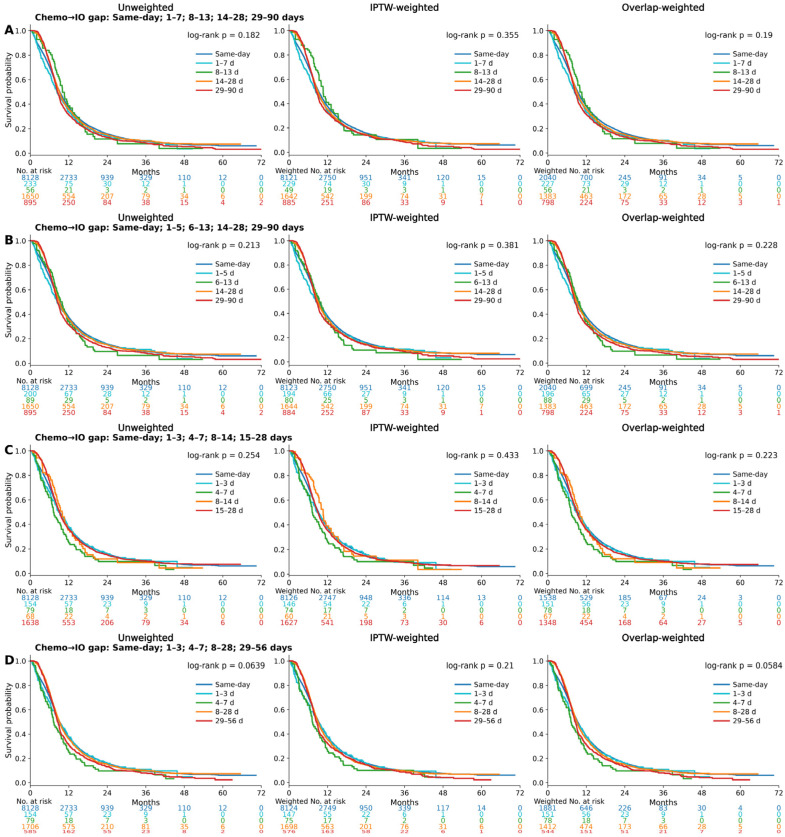
Survival analysis of ES-SCLC by different sequences of immunotherapy initiation of Chemo + IO: unweighted, IPTW, and OW Kaplan–Meier Curves. (**A**) Chemo + IO gap with time intervals (same day, 1–7 days, 8–13 days, 14–28 days, and 29–90 days). (**B**) Chemo + IO gap with time intervals (same day, 1–5 days, 6–13 days, 14–28 days, and 29–90 days). (**C**) Chemo + IO gap with time intervals (same day, 1–3 days, 4–7 days, 8–14 days, and 15–28 days). (**D**) Chemo + IO gap with time intervals (same day, 1–3 days, 4–7 days, 8–28 days, and 29–56 days).

Chemo + IO: This interval was defined as the time from the start of immunotherapy to the start of chemotherapy within the chemo–immunotherapy arm (Figure 4A–D, survival analysis by different sequences of immunotherapy initiation of Chemo + IO). Across multiple interval definitions—Figure 4A: same day, 1–7, 8–13, 14–28, and 29–90 days; Figure 4B: same day, 1–5, 6–13, 14–28, and 29–90 days; Figure 4C: same day, 1–3, 4–7, 8–14, and 15–28 days; and Figure 4D: same day, 1–3, 4–7, 8–28, and 29–56 days—survival analyses among patients with ES-SCLC receiving differing sequencing intervals between chemotherapy and immunotherapy did not reveal significant differences in OS. Log-rank *p*-values for all unweighted, IPTW-weighted, and OW survival curves remained consistently above 0.05, indicating that the chemotherapy and sequence of immunotherapy initiation do not significantly impact survival outcomes. These results suggest that the order of administration does not confer a survival benefit in ES-SCLC.

### 3.5. Survival in Different Sequences of CRT Plus Immunotherapy

**Figure 5:**
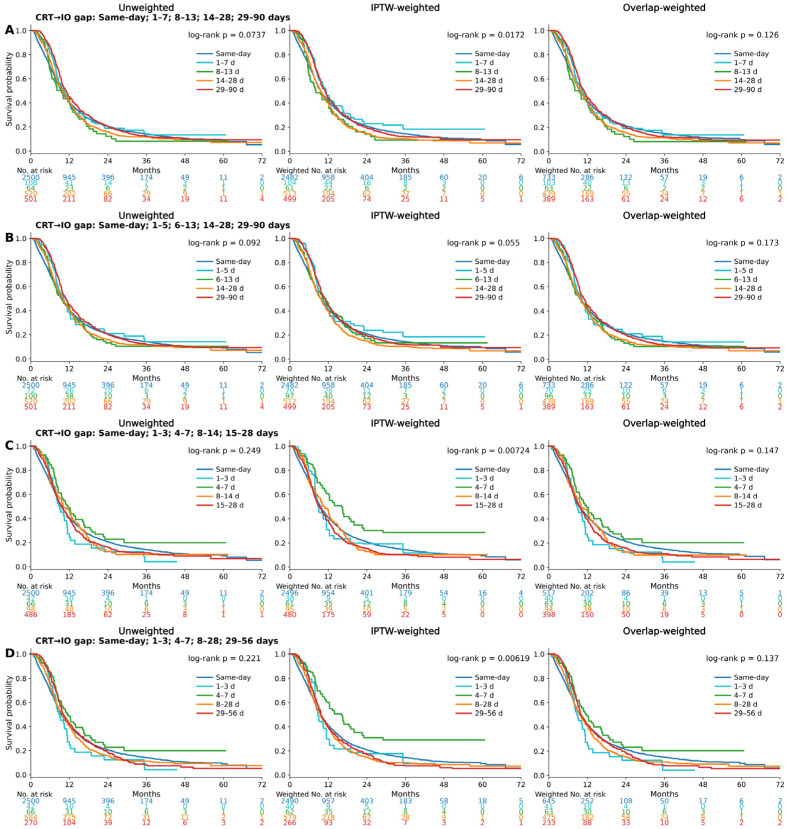
Survival analysis of ES-SCLC by different sequences of immunotherapy initiation of CRT + IO: unweighted, IPTW, and OW Kaplan–Meier Curves”. (**A**) CRT + IO gap with time intervals (same day, 1–7 days, 8–13 days, 14–28 days, and 29–90 days). (**B**) CRT + IO gap with time intervals (same day, 1–5 days, 6–13 days, 14–28 days, and 29–90 days). (**C**) CRT + IO gap with time intervals (same day, 1–3 days, 4–7 days, 8–14 days, and 15–28 days). (**D**) CRT + IO gap with time intervals (same day, 1–3 days, 4–7 days, 8–28 days, and 29–56 days).

**Figure A1:**
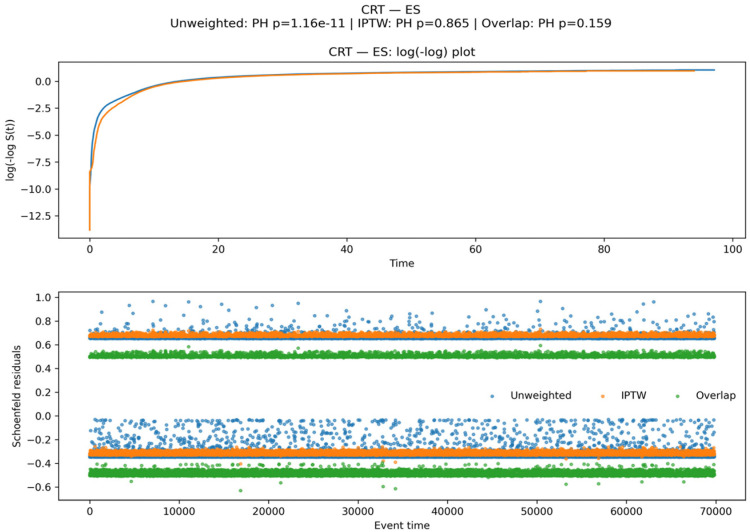
PH diagnostics for CRT vs. CRT + IO in ES: log(−log) survival and Schoenfeld residuals (unweighted, IPTW, and OW).

CRT + IO: This interval was defined as the time from the start of immunotherapy to the first day of concurrent CRT within the CRT + IO arm. In the CRT + IO cohort, stratification by various interval schemes (e.g., Figure 5A: same day, 1–7, 8–13, 14–28, and 29–90 days; Figure 5B: same day, 1–5, 6–13, 14–28, and 29–90 days) did not show a clear survival gradient. Upon refining the interval definitions (Figure 5C: same day, 1–3, 4–7, 8–14, and 15–28 days), the 4–7-day interval consistently demonstrated the most favorable survival outcomes. Further extension of the longer interval categories (Figure 5D: same day, 1–3, 4–7, 8–28, and 29–56 days) revealed a similar directional trend, with improved survival observed when immunotherapy was initiated 4–7 days after the start of CRT. The survival curve for the 4–7-day delay group showed the greatest separation from other groups, suggesting a potential survival advantage for initiating immunotherapy within this window. Although the magnitude of difference was modest, the 4–7-day group exhibited a trend toward improved outcomes compared to longer delays, implying that a shorter interval may enhance the therapeutic efficacy of immunotherapy. These findings were most pronounced under IPTW adjustment, whereas OW and unweighted estimates were attenuated toward nullification, likely due to residual confounding and reduced effective sample sizes in certain strata. PS diagnostics, including log(-log) survival curves and Schoenfeld residuals under unweighted, IPTW, and OW analyses, did not indicate major violations (Figure A1), supporting the validity of the sequence of immunotherapy initiation comparisons.

### 3.6. RMST Analysis of Survival in Different Sequences of CRT Plus Immunotherapy

**Figure A2:**
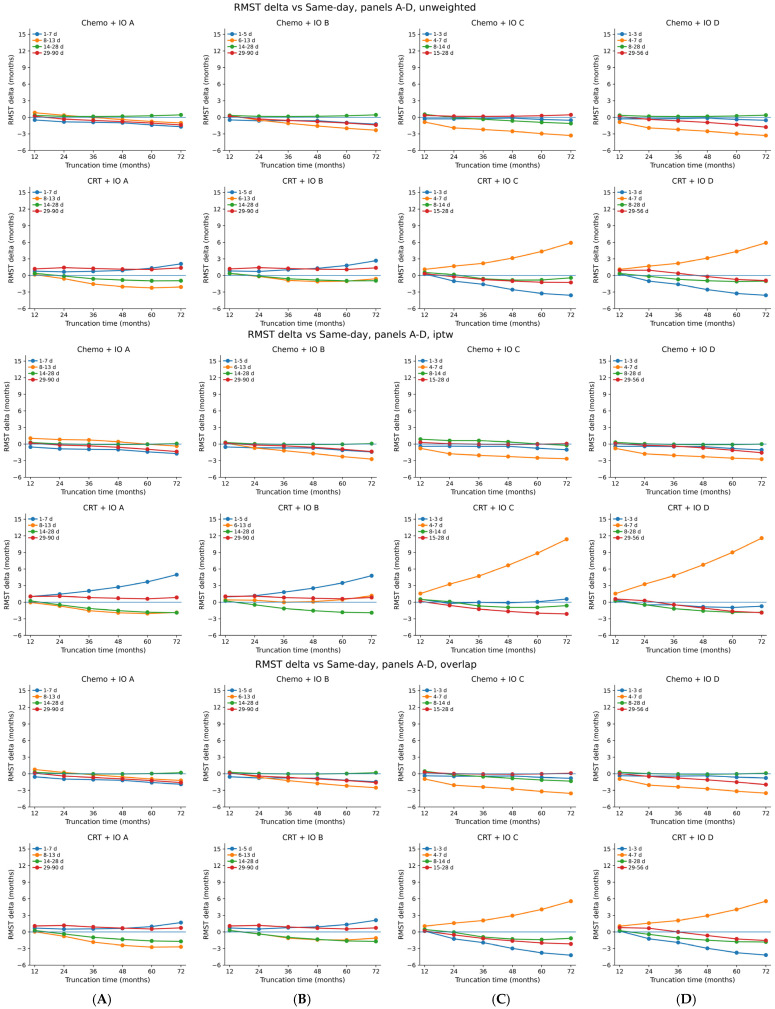
RMST differences across sequence of immunotherapy initiation groups in ES-SCLC. RMST differences in months across sequence strata in ES-SCLC, shown for Chemo + IO in the top row and CRT + IO in the bottom row. Columns A–D correspond to interval schemes A–D used to define sequence of immunotherapy initiation groups. RMST differences are calculated relative to the same-day reference group: (**A**) time intervals (1–7 days, 8–13 days, 14–28 days, and 29–90 days). (**B**) Time intervals (1–5 days, 6–13 days, 14–28 days, and 29–90 days). (**C**) Time intervals (1–3 days, 4–7 days, 8–14 days, and 15–28 days). (**D**) Time intervals (1–3 days, 4–7 days, 8–28 days, and 29–56 days).

To complement KM comparisons and provide an absolute measure of benefit, we summarized the sequence-related survival of immunotherapy initiation differences using RMST using same-day initiation as the reference. RMST results across alternative interval schemes A–D are provided in Figure A2.

Log-rank tests and Cox models assessed whether survival differed across sequence strata. To further quantify absolute benefit, we estimated RMST, which provides an interpretable measure of time gained over a prespecified truncation horizon. In Chemo + IO, RMST differences across sequence strata were consistently small across the evaluated truncation times and did not exhibit a coherent gradient across interval schemes. This pattern was broadly consistent across unweighted, IPTW-weighted, and OW analyses, supporting the conclusion that varying the sequence of immunotherapy initiation after chemotherapy did not yield a clinically meaningful or directionally consistent absolute survival gain in this cohort. In contrast, RMST patterns in CRT + IO were more informative and aligned with our central finding that the early post-CRT window may be clinically relevant. Specifically, when early initiation windows were defined to distinguish 1–3 days from 4 to 7 days, the 4–7-day group consistently showed a more favorable RMST profile than same-day initiation, with the clearest separation under IPTW weighting; over the prespecified truncation horizon, the corresponding absolute RMST gain about 10 months.

## 4. Discussion

In this population-based analysis using NCDB, we evaluated the impact of the sequence of immunotherapy initiation when added to chemotherapy or CRT on survival outcomes in patients with SCLC. In the overall cohort, the addition of immunotherapy to chemotherapy or CRT did not confer a significant survival advantage. However, among patients with ES-SCLC, immunotherapy was consistently associated with improved survival in both the chemotherapy and CRT subgroups (Chemo + IO: HR 0.58–0.67; CRT + IO: HR 0.80–0.83). In contrast, no survival benefit was observed with the addition of immunotherapy in LS-SCLC, a finding potentially influenced by the small sample size and baseline imbalances in the LS-SCLC subgroup receiving immunotherapy. These results are consistent with current treatment guidelines [10,25] recommending immunotherapy for ES-SCLC and align with evidence from pivotal phase III trials [6,26], demonstrating that the addition of adebrelimab or tislelizumab to etoposide–platinum (EP) chemotherapy significantly improves OS in ES-SCLC. These findings offer real-world support for the use of immunotherapy in this population.

Preclinical models suggest that antitumor efficacy may be modulated by the sequence of immunotherapy initiation relative to chemotherapy [27,28]. Real-world studies further indicate that the sequencing of immunotherapy in combination with chemotherapy or CRT can meaningfully influence survival outcomes. Previous research has shown that initiating immunotherapy within 30 days after chemotherapy is associated with significantly longer OS in patients with stage IV NSCLC compared to initiation between 31 and 60 days post-chemotherapy [29]. Another study demonstrated that patients with stage III NSCLC derive maximal survival benefit when immunotherapy is initiated within 10 to 12 weeks following chemoradiotherapy, with diminished effects observed when treatment is started beyond 12 weeks [30]. Nevertheless, the optimal sequence for immunotherapy initiation remains uncertain [31]. For SCLC, the effect of different immunotherapy initiation intervals on OS is not well defined, and large-scale, population-based assessments are lacking. To address this gap, our study employed multiple time intervals to evaluate whether the sequence of immunotherapy initiation, in combination with chemotherapy or CRT, influences OS in a large, real-world SCLC cohort.

Innate immunity provides an immediate, non-specific response to tumor-related injury or stress, triggering inflammation and the recruitment of immune cells. Both chemotherapy and radiotherapy are known to trigger ICD, leading to the release of tumor-associated antigens, damage-associated molecular patterns, and pro-inflammatory cytokines that activate innate immune responses within the first several days after treatment. This early innate immune activation is followed by antigen uptake and presentation by dendritic cells and the subsequent priming and clonal expansion of tumor-specific CD8^+^ T cells, a process that typically evolves over several days. Adaptive immunity develops more gradually through antigen presentation, leading to antigen-specific T-cell responses and immune memory that mediate sustained antitumor activity [15,32]. Substantial evidence supports the existence of an immune activation window following cytotoxic therapy; an immune checkpoint blockade administered during this transitional phase may be particularly effective by sustaining T-cell activation and amplifying adaptive immune responses [27].

Following chemotherapy, preclinical studies have demonstrated the rapid activation of both innate and adaptive immunity: in a Lewis lung carcinoma model [33], low-dose carboplatin increased tumor PD-L1 expression on day 2, peaking on day 3. Clinical data from patients with NSCLC receiving platinum-based chemotherapy showed that PD-1 expression on CD8^+^ T cells peaked on day 3 and returned to baseline by days 5–7; initiation of immunotherapy on day 3 after chemotherapy was associated with improved outcomes compared to concurrent administration [24]. In contrast, when radiotherapy is combined with immunotherapy, the peak of immune activation is delayed. In the ID8 model [34], low-dose irradiation (1 Gy) induced the significant infiltration of lymphocytes, NK cells, macrophages, and dendritic cells by day 5, with immunohistochemical confirmation on day 7, followed by a gradual decline in T-cell-mediated inflammation. Collectively, these findings suggest that the optimal sequence for immunotherapy initiation may differ depending on whether it follows chemotherapy or radiotherapy, reflecting the distinct kinetics of immune activation elicited by each modality.

Given that early differences in the combination sequence may significantly influence therapeutic efficacy, we evaluated multiple interval definitions (0 days, 1–7 days, 1–5 days, 1–3 days, 4–7 days, 8–14 days, and 15–28 days), each potentially exerting a distinct impact on clinical outcomes. To determine whether specific initiation windows influenced survival among patients with ES-SCLC who benefit from immunotherapy, we assessed both early intervals (same-day, 1–7 days, and 1–5 days) and later intervals (e.g., 8–14 days and 15–28 days). However, these broader time windows did not reveal a consistent survival benefit. We therefore refined the interval classifications to better distinguish the effects of very early initiation, comparing 1–3 days and 4–7 days relative to same-day initiation. Later intervals were grouped into two-week periods (8–14 or 15–28 days) or broader ranges (8–28, 29–56, or 29–90 days). Our analysis revealed that, in the CRT + IO cohort, the initiation of immunotherapy 4–7 days after CRT was associated with a favorable survival trend. In contrast, no comparable sequence of immunotherapy initiation-dependent pattern was observed in the Chemo + IO cohort. Longer initiation intervals did not confer meaningful survival differences in the Chemo + IO cohort.

Initiation of immunotherapy within a 4–7-day window after CRT may better align with the peak of adaptive immune activation than very early administration (e.g., same day or 1–3 days), which may predominantly coincide with innate immune signaling before sufficient antigen presentation has occurred. Conversely, the delayed initiation of immunotherapy beyond this window may miss the transient surge in tumor antigenicity and immune activation induced by CRT, thereby attenuating the potential synergistic effects of combined-modality treatment. Our results align with the concept of a post-CRT “immune activation window”. No significant timing differences were observed in the Chemo + IO cohort. The transient nature of chemotherapy-induced immune activation, combined with the limited temporal resolution of the NCDB, may have obscured subtle sequence-dependent effects. The findings are consistent with preclinical evidence indicating that checkpoint blockade is most effective when initiated at the peak of antigen-driven adaptive immune activation.

However, these findings should be interpreted in light of several important limitations inherent to NCDB. First, the NCDB does not provide detailed treatment information, including radiotherapy dose and fractionation, specific systemic therapy regimens, the number of chemotherapy cycles administered, PD-L1 expression status, treatment-related adverse events, or subsequent lines of therapy. Second, the number of patients in the CRT + IO cohort with precisely classifiable treatment intervals was modest. Subdivision into multiple sequences of immunotherapy initiation categories further reduced the effective sample sizes within individual strata. The record of NCDB comes from real-word clinical practice; the variety and diversity make it highly different, with selective clinical trial population records. Although we applied robust causal inference approaches, including IPTW and OW, residual confounding inherent to retrospective registry-based analyses cannot be fully excluded. The finding of the impact of immunotherapy sequencing in this cohort warrants further investigation in prospectively designed trials.

Collectively, our findings suggest that maximizing clinical benefit in ES-SCLC may depend not only on the addition of immunotherapy but also on aligning its initiation with the time-sensitive immune response elicited by CRT. These observations warrant prospective validation. Future randomized trials should define optimal initiation windows to determine when the immune response following CRT can be most effectively harnessed by checkpoint inhibition.

## 5. Conclusions

In conclusion, this real-world analysis suggests that immunotherapy is associated with prolonged survival in patients with ES-SCLC. CRT + IO is associated with improved OS, with a trend toward greater survival benefit when ICIs are initiated within 4–7 days after CRT. These findings indicate a potential association between the sequence of immunotherapy initiation and survival outcomes; prospective studies are needed to explore the optimal sequencing of immunotherapy.

## Figures and Tables

**Table 1:** Baseline characteristics (ES-SCLC): Chemo only vs. Chemo + IO.

Characteristic	Level	Chemo Only (*n* = 18,971)	Chemo + IO (*n* = 11,580)	SMD	IPTW SMD	Overlap SMD
Age	Mean (SD)	67.63 (9.18)	67.54 (8.94)	0.026	0.018	<0.001
Sex				0.015	0.002	<0.001
	Female	9341 (49.2%)	5921 (51.1%)			
	Male	9630 (50.8%)	5659 (48.9%)			
Race				0.01	0.007	0.004
	White	16,942 (89.3%)	10,364 (89.5%)			
	Black	1426 (7.5%)	833 (7.2%)			
	Others	603 (3.2%)	383 (3.3%)			
Insurance status				0.016	0.006	0.001
	Medicare	11,874 (62.6%)	7307 (63.1%)			
	Private insurance/managed care	4082 (21.5%)	2531 (21.9%)			
	Others	3015 (16.0%)	1742 (15.0%)			
Charlson–Deyo score				0.03	0.009	<0.001
	0	9996 (52.7%)	6323 (54.6%)			
	1	4686 (24.7%)	2802 (24.2%)			
	2	2235 (11.8%)	1334 (11.5%)			
	≥3	2054 (10.8%)	1121 (9.7%)			
Year of diagnosis				0.589	0.221	0.005
	2016	4935 (26.0%)	113 (1.0%)			
	2017	5010 (26.4%)	202 (1.7%)			
	2018	4009 (21.1%)	873 (7.5%)			
	2019	2030 (10.7%)	3290 (28.4%)			
	2020	1522 (8.0%)	3425 (29.6%)			
	2021	1465 (7.7%)	3677 (31.8%)			
Facility type				0.007	0.017	0.001
	Comprehensive community cancer program	7945 (41.9%)	4819 (41.6%)			
	Academic/research program	3885 (20.5%)	2391 (20.6%)			
	Integrated network cancer program	5390 (28.4%)	3328 (28.7%)			
	Community cancer program	1722 (9.1%)	1014 (8.8%)			
	Unknown	29 (0.2%)	28 (0.2%)			

**Table 2:** Baseline characteristics (ES-SCLC): CRT only vs. CRT + IO.

Characteristic	Level	CRT Only (*n* = 16,703)	CRT + IO (*n* = 8987)	SMD	IPTW SMD	Overlap SMD
Age	Mean (SD)	64.91 (8.82)	64.91 (8.72)	0.01	0.028	<0.001
Sex				0.038	0.002	<0.001
	Female	8546 (51.2%)	4516 (50.3%)			
	Male	8157 (48.8%)	4471 (49.7%)			
Race				0.01	0.009	0.006
	White	14,748 (88.3%)	8000 (89.0%)			
	Black	1533 (9.2%)	733 (8.2%)			
	Others	422 (2.5%)	254 (2.8%)			
Insurance status				0.019	0.014	0.001
	Medicare	8909 (53.3%)	4691 (52.2%)			
	Private insurance/managed care	4805 (28.8%)	2677 (29.8%)			
	Others	2989 (17.9%)	1619 (18.0%)			
Charlson–Deyo score				0.024	0.016	0.001
	0	9858 (59.0%)	5233 (58.2%)			
	1	4073 (24.4%)	2246 (25.0%)			
	2	1663 (10.0%)	899 (10.0%)			
	≥3	1109 (6.6%)	609 (6.8%)			
Year of diagnosis				0.601	0.095	0.005
	2016	5193 (31.1%)	142 (1.6%)			
	2017	4857 (29.1%)	196 (2.2%)			
	2018	3457 (20.7%)	823 (9.2%)			
	2019	1397 (8.4%)	2608 (29.0%)			
	2020	935 (5.6%)	2665 (29.7%)			
	2021	864 (5.2%)	2553 (28.4%)			
Facility type				0.01	0.023	0.001
	Comprehensive community cancer program	6985 (41.8%)	3487 (38.8%)			
	Academic/research program	4743 (28.4%)	2827 (31.5%)			
	Integrated network cancer program	3266 (19.6%)	1861 (20.7%)			
	Community cancer program	1660 (9.9%)	780 (8.7%)			
	Unknown	49 (0.3%)	32 (0.4%)			

Notes and statistical methods: (1) Descriptive, unweighted. YEAR_OF_DIAGNOSIS is treated as an ordered categorical variable (2016–2021). (2) For continuous variables (age), Kruskal–Wallis test is used across the four groups. (3) For categorical variables, Pearson’s χ^2^ test is used. If a 2 × 2 table has any expected cell <5, Fisher’s exact test is used instead. (4) *p*-values are shown on the variable header row only (levels below omit *p*). Display rules: *p* < 0.001 shown as “<0.001”; otherwise, three decimals (e.g., 0.012).(5) No multiplicity adjustment; interpret *p*-values as exploratory.

## Data Availability

The datasets presented in this study can be found in NCDB. The datasets generated and/or analyzed during the current study are available from the corresponding authors upon reasonable request.

## Abbreviations

The following abbreviations are used in this manuscript:

SCLC	Small-cell lung cancer
ES-SCLC	Extensive-stage small-cell lung cancer
LS-SCLC	Limited-stage small-cell lung cancer
NSCLC	Non-small cell lung cancer
NCDB	National Cancer Database
PFS	Progression-free survival
OS	Overall survival
CRT	Chemoradiotherapy
Chemo	Chemotherapy
IO	Immunotherapy
ICIs	Immune checkpoint inhibitors
PD-1	Programmed cell death protein 1
PD-L1	Programmed death-ligand 1
ICD	Immunogenic cell death
EP	Etoposide-platinum
PS	Propensity scores
IPTW	Inverse probability of treatment weighting
OW	Overlap weights
ASMD	Absolute standardized mean differences
PH	Proportional hazards
HR	Hazard ratio
CIs	Confidence intervals
KM	Kaplan-Meier
RMST	Restricted mean survival time
SD	Standard deviation

## References

[1] Megyesfalvi Z., Gay C.M., Popper H., Pirker R., Ostoros G., Heeke S., Lang C., Hoetzenecker K., Schwendenwein A., Boettiger K. (2023). Clinical insights into small cell lung cancer: Tumor heterogeneity, diagnosis, therapy, and future directions. CA A Cancer J. Clin..

[2] Kim S.Y., Park H.S., Chiang A.C. (2025). Small Cell Lung Cancer: A Review. JAMA.

[3] Zhai X., Zhang Z., Chen Y., Wu Y., Zhen C., Liu Y., Lin Y., Chen C. (2025). Current and future therapies for small cell lung carcinoma. J. Hematol. Oncol..

[4] Simpson K.L., Rothwell D.G., Blackhall F., Dive C. (2025). Challenges of small cell lung cancer heterogeneity and phenotypic plasticity. Nat. Rev. Cancer.

[5] Thomas A., Mohindroo C., Giaccone G. (2025). Advancing therapeutics in small-cell lung cancer. Nat. Cancer.

[6] Cheng Y., Fan Y., Zhao Y., Huang D., Li X., Zhang P., Kang M., Yang N., Zhong D., Wang Z. (2024). Tislelizumab Plus Platinum and Etoposide Versus Placebo Plus Platinum and Etoposide as First-Line Treatment for Extensive-Stage SCLC (RATIONALE-312): A Multicenter, Double-Blind, Placebo-Controlled, Randomized, Phase 3 Clinical Trial. J. Thorac. Oncol..

[7] Cheng Y., Han L., Wu L., Chen J., Sun H., Wen G., Ji Y., Dvorkin M., Shi J., Pan Z. (2022). Effect of First-Line Serplulimab vs Placebo Added to Chemotherapy on Survival in Patients With Extensive-Stage Small Cell Lung Cancer: The ASTRUM-005 Randomized Clinical Trial. JAMA.

[8] Liu S.V., Reck M., Mansfield A.S., Mok T., Scherpereel A., Reinmuth N., Garassino M.C., De Castro C.J., Califano R., Nishio M. (2021). Updated Overall Survival and PD-L1 Subgroup Analysis of Patients With Extensive-Stage Small-Cell Lung Cancer Treated With Atezolizumab, Carboplatin, and Etoposide (IMpower133). J. Clin. Oncol..

[9] Paz-Ares L., Dvorkin M., Chen Y., Reinmuth N., Hotta K., Trukhin D., Statsenko G., Hochmair M.J., Ozguroglu M., Ji J.H. (2019). Durvalumab plus platinum-etoposide versus platinum-etoposide in first-line treatment of extensive-stage small-cell lung cancer (CASPIAN): A randomised, controlled, open-label, phase 3 trial. Lancet.

[10] Dingemans A.C., Früh M., Ardizzoni A., Besse B., Faivre-Finn C., Hendriks L.E., Lantuejoul S., Peters S., Reguart N., Rudin C.M. (2021). Small-cell lung cancer: ESMO Clinical Practice Guidelines for diagnosis, treatment and follow-up(☆). Ann. Oncol. Off. J. Eur. Soc. Med. Oncol..

[11] Paz-Ares L., Borghaei H., Liu S.V., Peters S., Herbst R.S., Stencel K., Majem M., Şendur M.A.N., Czyżewicz G., Caro R.B. (2025). Efficacy and safety of first-line maintenance therapy with lurbinectedin plus atezolizumab in extensive-stage small-cell lung cancer (IMforte): A randomised, multicentre, open-label, phase 3 trial. Lancet.

[12] Cheng Y., Spigel D.R., Cho B.C., Laktionov K.K., Fang J., Chen Y., Zenke Y., Lee K.H., Wang Q., Navarro A. (2024). Durvalumab after Chemoradiotherapy in Limited-Stage Small-Cell Lung Cancer. N. Engl. J. Med..

[13] Zhang Z., Liu X., Chen D., Yu J. (2022). Radiotherapy combined with immunotherapy: The dawn of cancer treatment. Signal Transduct. Target. Ther..

[14] Kwon M., Jung H., Nam G.H., Kim I.S. (2021). The right Timing, right combination, right sequence, and right delivery for Cancer immunotherapy. J. Control. Release.

[15] Galluzzi L., Guilbaud E., Schmidt D., Kroemer G., Marincola F.M. (2024). Targeting immunogenic cell stress and death for cancer therapy. Nat. Rev. Drug Discov..

[16] Ghiringhelli F., Rebe C. (2024). Using immunogenic cell death to improve anticancer efficacy of immune checkpoint inhibitors: From basic science to clinical application. Immunol. Rev..

[17] Rausch L., Kallies A. (2025). Molecular Mechanisms Governing CD8 T Cell Differentiation and Checkpoint Inhibitor Response in Cancer. Annu. Rev. Immunol..

[18] Huo Y., Wang D., Yang S., Xu Y., Qin G., Zhao C., Lei Q., Zhao Q., Liu Y., Guo K. (2024). Optimal timing of anti-PD-1 antibody combined with chemotherapy administration in patients with NSCLC. J. Immunother. Cancer.

[19] Jackson S.S., Han X., Mao Z., Nogueira L., Suneja G., Jemal A., Shiels M.S. (2021). Cancer Stage, Treatment, and Survival Among Transgender Patients in the United States. J. Natl. Cancer Inst..

[20] Kamarajah S.K., Sonnenday C.J., Cho C.S., Frankel T.L., Bednar F., Lawrence T.S., Nathan H. (2021). Association of Adjuvant Radiotherapy With Survival After Margin-negative Resection of Pancreatic Ductal Adenocarcinoma: A Propensity-matched National Cancer Database (NCDB) Analysis. Ann. Surg..

[21] Ow T.J., Mehta V., Kim S., Vakil M., Friedmann P., In H. (2022). Evaluation of Survival and Postoperative Radiation Among Patients with Advanced Medullary Thyroid Carcinoma: An Analysis of the National Cancer Database. Ann. Surg. Oncol..

[22] Boffa D.J., Rosen J.E., Mallin K., Loomis A., Gay G., Palis B., Thoburn K., Gress D., Mckellar D.P., Shulman L.N. (2017). Using the National Cancer Database for Outcomes Research: A Review. JAMA Oncol..

[23] Thomas L.E., Li F., Pencina M.J. (2020). Overlap Weighting: A Propensity Score Method That Mimics Attributes of a Randomized Clinical Trial. JAMA.

[24] Castelo-Branco L., Pellat A., Martins-Branco D., Valachis A., Derksen J., Suijkerbuijk K., Dafni U., Dellaporta T., Vogel A., Prelaj A. (2023). ESMO Guidance for Reporting Oncology real-World evidence (GROW). Ann. Oncol..

[25] Ganti A., Loo B.W., Bassetti M., Blakely C., Chiang A., D’Amico T.A., D’Avella C., Dowlati A., Downey R.J., Edelman M. (2021). Small Cell Lung Cancer, Version 2.2022, NCCN Clinical Practice Guidelines in Oncology. J. Natl. Compr. Cancer Netw..

[26] Wang J., Zhou C., Yao W., Wang Q., Min X., Chen G., Xu X., Li X., Xu F., Fang Y. (2022). Adebrelimab or placebo plus carboplatin and etoposide as first-line treatment for extensive-stage small-cell lung cancer (CAPSTONE-1): A multicentre, randomised, double-blind, placebo-controlled, phase 3 trial. Lancet Oncol..

[27] Williamson C.W., Sherer M.V., Zamarin D., Sharabi A.B., Dyer B.A., Mell L.K., Mayadev J.S. (2021). Immunotherapy and radiation therapy sequencing: State of the data on timing, efficacy, and safety. Cancer-Am. Cancer Soc..

[28] Zhu C., Shi Y., Li Q., Luo L., Li X., Luo Z., Lu Y., Zhang J., Jiang M., Qin B. (2022). Rational administration sequencing of immunochemotherapy elicits powerful anti-tumor effect. J. Control. Release.

[29] Vazquez-Urrutia J.R., Greenberg M., Zhu J., Takamori S., Komiya T. (2024). Prognostic Implications of Timing of Immunotherapy in Stage IV Non-Small Cell Lung Cancer. World J. Oncol..

[30] Pichert M.D., Canavan M.E., Maduka R.C., Li A.X., Ermer T., Zhan P.L., Kaminski M., Udelsman B.V., Blasberg J.D., Park H.S. (2022). Immunotherapy After Chemotherapy and Radiation for Clinical Stage III Lung Cancer. JAMA Netw. Open.

[31] Sun Q., Hong Z., Zhang C., Wang L., Han Z., Ma D. (2023). Immune checkpoint therapy for solid tumours: Clinical dilemmas and future trends. Signal Transduct. Target. Ther..

[32] Catanzaro E., Beltran-Visiedo M., Galluzzi L., Krysko D.V. (2025). Immunogenicity of cell death and cancer immunotherapy with immune checkpoint inhibitors. Cell. Mol. Immunol..

[33] Zhou L., Xu Q., Huang L., Jin J., Zuo X., Zhang Q., Ye L., Zhu S., Zhan P., Ren J. (2021). Low-dose carboplatin reprograms tumor immune microenvironment through STING signaling pathway and synergizes with PD-1 inhibitors in lung cancer. Cancer Lett..

[34] Herrera F.G., Ronet C., Ochoa D.O.M., Barras D., Crespo I., Andreatta M., Corria-Osorio J., Spill A., Benedetti F., Genolet R. (2022). Low-Dose Radiotherapy Reverses Tumor Immune Desertification and Resistance to Immunotherapy. Cancer Discov..

